# Dual-Branch Cross-Fusion Normalizing Flow for RGB-D Track Anomaly Detection

**DOI:** 10.3390/s25082631

**Published:** 2025-04-21

**Authors:** Xiaorong Gao, Pengxu Wen, Jinlong Li, Lin Luo

**Affiliations:** School of Physical Science and Technology, Southwest Jiaotong University, Chengdu 610031, China; gxrr@vip.163.com (X.G.); wenpx@my.swjtu.edu.cn (P.W.); happyluolin@vip.163.com (L.L.)

**Keywords:** anomaly detection, normalizing flow, RGB-D fusion

## Abstract

With the ease of acquiring RGB-D images from line-scan 3D cameras and the development of computer vision, anomaly detection is now widely applied to railway inspection. As 2D anomaly detection is susceptible to capturing condition, a combination of depth maps is now being explored in industrial inspection to reduce these interferences. In this case, this paper proposes a novel approach for RGB-D anomaly detection called Dual-Branch Cross-Fusion Normalizing Flow (DCNF). In this work, we aim to exploit the fusion strategy for dual-branch normalizing flow with multi-modal inputs to be applied in the field of track detection. On the one hand, we introduce the mutual perception module to acquire cross-complementary prior knowledge in the early stage. On the other hand, we exploit the effectiveness of the fusion flow to fuse the dual-branch of RGB-D inputs. We experiment on the real-world Track Anomaly (TA) dataset. The performance evaluation of DCNF on TA dataset achieves an impressive AUROC score of 98.49%, which is 3.74% higher than the second-best method.

## 1. Introduction

The popularization of high-speed trains has made this study convenient for us. With the increase in running times, regular track inspection greatly affects the safety of the train. Early industrial inspection methods were usually based on manual inspection. However, the efficiency of this approach is low and it fails to meet the increasing demand for inspection [1,2]. With the development of deep learning, computer vision has shown excellent performance in automatic industrial detection [3,4], and deep learning is gradually being applied to high-speed train detection. With a line-scan 3D camera mounted on the bottom of the inspection train, we can automatically collect the track images, and deep learning helps us automate anomaly detection.

In anomaly detection task, it is hard to build a supervised model due to the lack of labeled anomaly data that present significant challenges in industrial detection [5,6]. In this case, most works utilize the distribution of normal data to build models, and others that do not match the normal distribution will be considered as an anomaly. Recent works focus on anomaly detection at the 2D level, usually using the color information of the image to determine whether there is an anomaly [7,8]. However, in industrial detection tasks, the use of 2D-only images might cause false positives or missed detection. In this paper, we extend 2D anomaly detection to 3D, in combination with depth maps, to take advantage of their shape information.

In industrial applications, the acquisition of depth maps by line-scan 3D cameras has obvious advantages over the acquisition of point clouds by 3D cameras. Firstly, the line-scan 3D camera has the capacity for fast scanning, which can simultaneously obtain the RGB images and depth maps of the whole object in a short time. Secondly, the line-scan 3D camera provides the depth information of each pixel, which ensures high-precision measurement and detection results. In addition, for the shape scanning of moving objects, line-scan 3D cameras are more convenient and suitable for scenes that require real-time monitoring or measurement. At the same time, due to the simplicity of depth map data processing, it can be directly applied to computer vision algorithms, which greatly simplifies the subsequent data processing flow. In this case, the objective of this paper is to build an RGB-D anomaly detection network for RGB images and depth maps, with the aim of enhancing the efficiency and performance of industrial detection.

The fusion strategies of RGB images and depth maps can be categorized as either early fusion or late fusion [9]. In the case of early fusion, the RGB images and depth maps are concatenated to form a four- or six-channels input. The late fusion typically establishes two branches for RGB images and depth maps, subsequently integrating their high-level features. The present paper introduces a novel cross-fusion strategy for RGB-D anomaly detection called Dual-Branch Cross-Fusion Normalizing Flow (DCNF). Inspired by [10], we adopt the concept of muti-scale normalizing flow, and set up a dual-branch normalizing flow framework to process the RGB images and depth maps separately. Besides this, we propose the Mutual Perception module (MP) to facilitate early-stage information exchange between the two branches, followed by the fusion of the two branches through a Fusion Flow in order to derive the anomaly score.

We evaluate the performance of DCNF on the Track Anomaly dataset (TA), which is collected from actual track anomalies, and achieves an advanced accuracy, while the results demonstrate the effectiveness of DCNF.

The contributions of this paper are outlined as follows:

1. Introduction of a novel RGB-D anomaly detection method in combination with depth maps to take advantage of the shape information;
2. Proposal of a dual-branch normalizing flow with cross-fusion strategy that fuses RGB images and depth maps;
3. Introduction of the Mutual Perception module (MP) and Fusion Flow to improve the compatibility of RGB-D data;
4. Achievement of advanced accuracy in TA dataset.

The remainder of this article is structured as follows: Section 2 provides a concise overview of prior research relevant to our methodology. The proposed DCNF is elaborated in detail in Section 3. Section 4 presents extensive experiments and analyses conducted to evaluate the effectiveness of our approach. Finally, in Section 5, a comprehensive conclusion summarizing the key findings of this article is provided.

## 2. Materials and Methods

### 2.1. 2D Anomaly Detection

Since the proposal of the MVTec AD [11], it has become a benchmark of 2D anomaly detection, and 2D anomaly detection has received more attention. Most of the works focus on the defect-free data, which are considered as unsupervised tasks.

#### 2.1.1. Reconstruction-Based Methods

The reconstruction-based methods are widely based on modeling the normal distribution, and are used reconstruct the unseen data. This model could generate defect-free data well, which accord with the normal distribution and fail to reconstruct the anomaly [12]. Autoencoder (AE) is a typical neural network model. The model realizes the feature learning and information reconstruction of input data by constructing a symmetric structure of “coding–decoding”. Specifically, the encoder module maps the high-dimensional input data to the low-dimensional potential space through nonlinear transformation, and the decoder module is responsible for restoring the compressed feature vector to the original data form. GANomaly is a classic reconstruction-based method [13] that leverages the training of generative adversarial networks (GANs) to model the distribution of normal data and subsequently reconstruct input data using GANs. However, these methods heavily rely on the quality and quantity of training data, which may limit their performance when applied to small datasets.

#### 2.1.2. Embedding Similarity-Based Methods

One of the other categories is embedding similarity-based methods, wherein the ImageNet pretrained feature extractor is employed for extracting image vectors [14]. Cho et al. introduced SPADE [8], which incorporates a multiresolution feature pyramid and employs k-nearest neighbor (kNN) techniques to effectively exploit deep pretrained features. Defard et al. introduced PaDiM [15], which maps the features from the pre-trained network to a Gaussian distribution and builds the corresponding memory library. Despite the use of patch anomaly insertion techniques, these methods still encounter difficulties in detecting minute or intricate anomalies. Therefore, these approaches may not be suitable for tasks involving the segmentation of fine anomalies [16].

#### 2.1.3. Normalizing Flows-Based Methods

In recent years, normalizing flows (NFs) have been gaining increasing attention by virtue of their efficient reversible transformation. Under the same principle as Real-NVP [17], the NFs first map the original distribution into the hidden space and reflect it back to the original sample distribution through the invertible function. With the rise of computer vision, NFs are gradually being used in the field of anomaly detection. DifferNet [18] leverages NFs to exploit the descriptive features extracted by convolutional neural networks to estimate their density. Following this, Gudovskiy et al. proposed CFlow [19], a more computationally and memory-efficient anomaly detection framework based on a conditional NFs framework. This structure not only retains the likelihood estimation capability of DifferNet, but also realizes the explicit probability judgment of the coding features by introducing conditional normalization flow. Specifically, CFlow uses a convolutional neural network (CNN) as the encoder in the feature extraction stage, and uses a reversible normalized flow network in the decoding stage. Through this design, anomaly localization at the pixel level is realized.

### 2.2. RGB-D Fusion Strategies

The early RGB-D fusion strategies tended to utilize the hand-craft feature extractors and fuse the RGB images and depth maps [20,21]. Despite the effectiveness of hand-craft features, the low-level features still lack generalizability. With the increasing number of works on deep-learning, the question emerges of how to leverage the multi-modal correlations and multi-level information.

Currently, RGB-D fusion strategies are being widely investigated in saliency object detection tasks. Existing RGB-D fusion strategies are broadly categorized into early fusion and late fusion. Early fusion crudely concatenates RGB images and depth maps into four- or six-channel inputs [22,23]. Late fusion usually builds two branches that process RGB images and depth maps separately, and fuses the processed high-level features at a later stage [24,25]. Wu et al. [26] introduced a layer-wise attention and trident spatial attention mechanisms to weigh up the early and late fusion of RGB and depth features and address depth dissonance. Inspired by the salient object detection task, the goal of our paper is to build an RGB-D fusion strategy that effectively incorporates the depth maps to take advantage of the shape information.

## 3. Method

**Figure 1:**
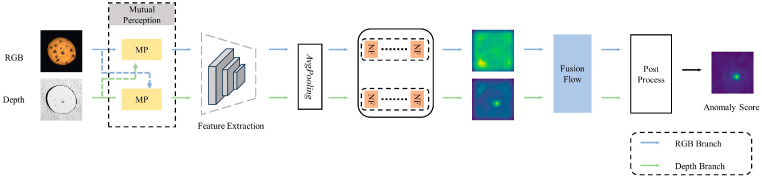
Structural image of DCNF network: Illustrating Mutual Perception, feature extraction, pooling layer, NF Blocks, fusion flow and post-processing for RGB-depth data-driven anomaly detection.

The proposed method of Dual-Branch Cross-Fusion Normalizing Flow (DCNF) is a dual-branch normalizing flow strategy with cross-fusion. As shown in Figure 1, we first input RGB images and depth maps into different branches of the dual-branch network. Subsequently, the Mutual Perception module (MP) allows each branch to get complementary information about the other branch, followed by a pretrained extractor to extract the cross-complementary feature. The dual-branch feature maps are then sent into the fully convolutional normalizing flow string, and we exchange the cross-complementary feature through Fusion Flow.

### 3.1. Mutual Perception Module (MP)

**Figure 2:**
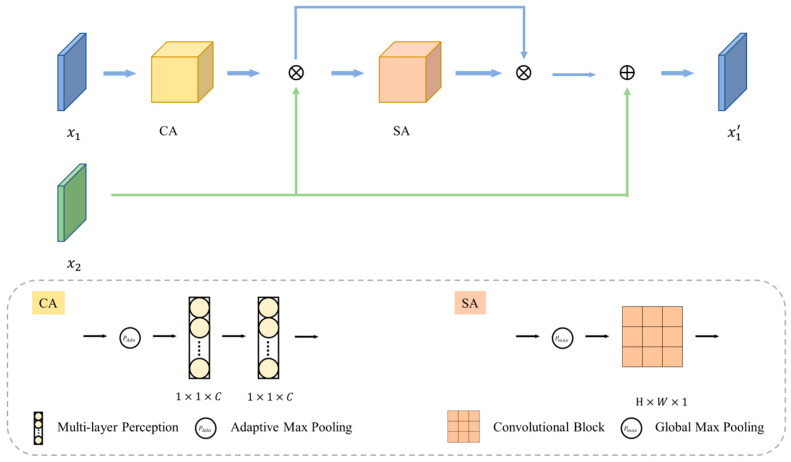
The overall architecture of the Mutual Perception module. The CA denotes the Channel Attention module, and the SA denotes the Spatial Attention module.

The fusion of RGB and depth features encounters two primary challenges. One pertains to the compatibility issue arising from muti-modal differences, while the other concerns the presence of duplications and interference in depth features with poor quality. Building upon [27], we introduced the MP to enhance the compatibility among multi-modal features and extract informative cues from deep features. The architecture of MP is illustrated in Figure 2.

Specifically, $x_{1}$ and $x_{2}$ denote the input of the RGB and depth branches, respectively. We define $x_{1}$ as the primary input and $x_{2}$ as the secondary input. The objective is to enable $x_{1}$ to incorporate complementary information from $x_{2}$, thereby acquiring cross-complementary prior knowledge. The calculation process is delineated as follows:

(1) $$F_{MP}(x_{1})=CA(x_{1})⨂x_{2}\;\begin{array}{c} x_{1}^{\prime }=SA\left(F_{MP}\left(x_{1}\right)\right)⨂F_{MP}\left(x_{1}\right)⨁x_{2} \end{array}$$

where CA(⋅) denotes the Channel Attention module and SA(⋅) denotes the Spatial Attention module. CA(⋅) aims to utilize the inter-channel relations between the depth and RGB features, and SA(⋅) determines the position that carries the depth features. The CA(⋅) and SA(⋅) are implemented as

(2) $$CA\left(x_{i}\right)={M(P}_{Ada}(x_{i}))\;\begin{array}{c} SA\left(x_{i}\right)=Conv\left(P_{max}\left(x_{i}\right)\right) \end{array}$$

where M(⋅) denotes the multi-layer perception, $P_{Ada}(\cdot )$ represents the adaptive max pooling applied to each branch of features and $P_{max}(\cdot )$ signifies the global max pooling applied to each point in the feature map across the channel axis.

### 3.2. Feature Extraction

The study conducted by Schirrmeister et al. [28] has demonstrated that the feature extractors, which are pre-trained on the ImageNet dataset, effectively capture the representative distribution of the data. Relevant features for anomaly detection can be extracted by pretrained CNNs [8]. The third layer of Wide-Resnet50-2 is utilized in this paper for feature extraction, enabling the inclusion of more intricate spatial details. While pixel-level anomaly localization tasks inherently prioritize spatial structure over semantic understanding, image-level anomaly detection tasks rely heavily on localized anomaly regions, making detail-rich feature maps more suitable. In order to minimize computational expenses, we incorporate a pooling layer following feature extraction to down-sample the extracted features. The features can be expressed as:

(3) $$y_{i}=AvgPool\left({E(x}_{i}^{\prime })\right)$$

where E(⋅) denotes the CNNs feature extraction and AvgPool(⋅) denotes the average pooling layer.

### 3.3. Fully Convolutional Normalizing Flow

**Figure 3:**
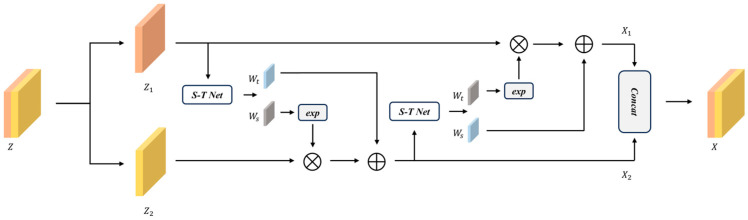
The architecture of the fully convolutional normalizing flow block.

The DCNF model consists of two independent fully convolutional normalizing flow branches with distinct weights. Each branch of a normalizing flow consists of n flow blocks, which encode the feature map $y_{i}$ and transform it into the latent feature map $z_{i}=f_{1\;or\;2}(y_{i})$. The dual normalizing flow branches share the same architecture as is illustrated in Figure 3; we use 3×3 convolution to construct the normalizing flow branches, which automatically capture the spatial context information that is ignored in [19]. Specifically, the S-T Net is used to calculate the scale weights $w_{i}^{s}$ and shift weights $w_{i}^{t}$,

(4) $${Net}_{S-T}(\cdot )={Conv}_{3\times 3}\circ RELU\circ LN\circ {Conv}_{3\times 3}\left(\cdot \right)$$

where ${Conv}_{3\times 3}(\cdot )$ represents the 3×3 convolution, RELU refers to the ReLU activation function and LN presents the layer normalization. The incorporation of LN enhances process stability and elevates model performance.

The bijective reversible normalizing flow can be implemented by following

(5) $$forward:\left\{\begin{array}{c} X_{1}=Z_{1}\\ X_{2}=Z_{2}exp⨀\left(W_{s}\left(Z_{1}\right)\right)+W_{t}(Z_{1}) \end{array}\right.\;reverse:\left\{\begin{array}{c} Z_{1}=X_{1}\\ Z_{2}=\left(X_{2}-W_{t}\left(X_{1}\right)\right)⨀exp(\left(-W_{s}\left(X_{1}\right)\right)) \end{array}\right.$$

where $W_{s}$ and $W_{t}$ generate the scale weights $w_{i}^{s}$ and shift weights $w_{i}^{t}$. The Jacobian determinant could be calculated as

(6) $$log\left|\det\left(J_{f^{-1}}\right)\right|=\sum W_{s}(X_{1})$$

### 3.4. Fusion Flow

The initial stage of network operation involves a simple interaction between the two branches. However, the primary concern lies in effectively integrating the features from both branches. In this case, we propose fusion flow to fuse the feature, followed by the normalizing flow. The proposed fusion flow is able to capture different parts of information using different branches of perception.

The fusion flow first fuses the dual-branch inputs $a_{1},a_{2}$ and then trains the scale weights $w_{i}^{s}$ and shift weights $w_{i}^{t}$ of the fusion flow:

(7) $$\left(z_{1}^{fuse},\;z_{2}^{fuse}\right)=g^{fuse}(a_{1},a_{2})$$

**Figure 4:**
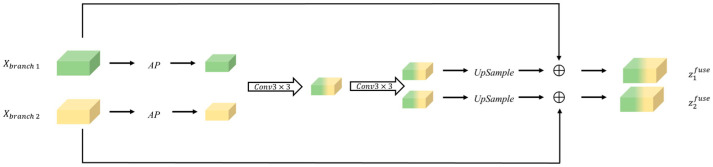
The architecture of the fusion flow module.

The $g^{fuse}(\cdot )$ is depicted in Figure 4; the fusion flow integrates the branches by means of an average pooling layer and establishes their connection. Subsequently, the two branches are merged using a 3×3 full convolution. The resulting feature map is then restored to its original size through up-sampling. Finally, the fused features are combined with their respective inputs in an element-wise addition process to obtain the fused inputs $z_{1}^{fuse}$ and $z_{2}^{fuse}$.

### 3.5. Learning Objective and Post Processing

Similar to [29], our training objective function is derived as:

(8) $$loss=\sum_{i=1}^{2}\frac{{\left\|z_{i}^{fuse}\right\|}_{2}^{2}}{2}-log\left|\det\left(J_{f^{-1}}\right)\right|$$

where the ${\left\|\cdot \right\|}_{2}^{2}$ represents the squared $l_{2}$-norm of a vector x in n-dimensional Euclidean space and $\left|\det\left(J_{f^{-1}}\right)\right|$ denotes the Jacobian determinants of the fully convolutional normalizing flows and fusion flows.

The main objective of the anomaly task is to locate the anomaly. Previous works have typically mapped z to a latent space and generated an anomaly score. In the paper, we define the anomaly scores for each local position. Specifically, we upsample z back to the original size and compute its squared mean to get the anomaly score for each location, as follows:

(9) $${Score}_{i}=\mathrm{mean}\left({z_{i}^{fuse}}^{2},dim=1\right)$$

Different from previous methods that either take the maximum score in a pixel-by-pixel anomaly score map or take the average score as the global anomaly score [19,20], our DCNF takes the average of the maximum anomaly scores of TopK in the spatial dimension as the global anomaly score, as follows:

(10) $$S=\frac{1}{K}\sum_{i=1}^{K}TopK\left({Score}_{i}\right)$$

where ${Score}_{i}$ represents the anomaly score of each pixel, and maximum and average are special cases of solutions when the K is set as 1 or $H_{img}\times W_{img}$.

## 4. Experiment

### 4.1. Datasets and Metrics

**Figure 5:**
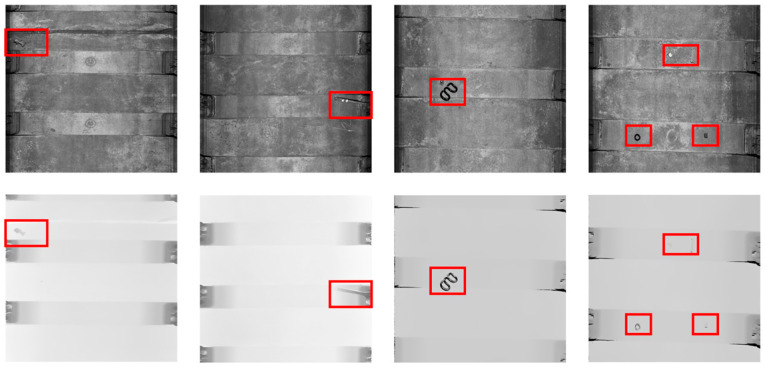
Examples of normal and anomaly images in the TA dataset.

Since there is no public dataset for the RGB-D anomaly detection task, we have collected a real-world Track-Anomaly dataset (TA), which includes foreign objects on rail tracks as well as RGB images and depth maps for each data set. The TA dataset contains 580 defect-free images in the train set and 330 images in the test set, which contains both defect and defect-free images. Figure 5 shows examples of the images in the TA dataset. The majority of anomalies in track anomaly detection are foreign objects, and the TA dataset contains both large-scale and small-scale foreign objects, which we consider suitable for industrial track detection scenarios.

Similar to the current anomaly detection methods, we here assess the effectiveness of our proposed DCNF using the Area Under Receiver Operator Curve (AUROC) [30], and subsequently evaluate its performance at the point of anomaly detection. Besides this, we compute the recall as the detection rate of anomalies for industrial evaluation.

### 4.2. Experimental Details

We utilize the output of layer 3 with a channel of 1024 from wide-resnet-50 (WRN-50) pretrained using ImageNet as the feature extractor for extracting higher-level image features. To reduce the computational effort, we input the extracted features into the average pooling layer for down-sampling. In fully convolutional normalizing flow blocks, we set the number of normalizing flow blocks to n=8 for all the experiments. We resize the images to (512×512) for the TA dataset. In our implementation, we adopt the Adam optimizer with the learning rate of $1\times 10^{-4}$. In the post-processing stage, we set the TopK to $0.05\times H_{img}\times W_{img}$. We train the DCNF for 120 epochs on the TA dataset. These experiments are carried out with an NVIDIA RTX 3090 24G GPU, which is manufactured by NVIDIA and procured from China.

Specifically, we initialize the feature extractor using the initial weights pretrained from ImageNet and freeze its parameters during training. All other modules use manual seed 3407 to initialize the weights and update them during training.

### 4.3. Quantitative Comparison

**Figure 6:**
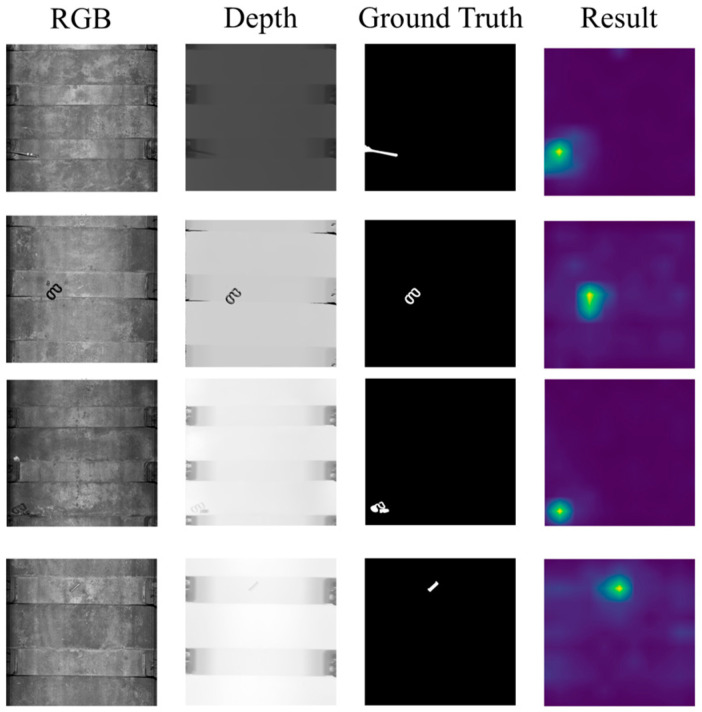
Examples of test results on the TA dataset. From left to right are RGB images, depth maps, Ground Truths and the visualization of the results.

The performance of DCNF is compared with the performances of prior methods including GANomaly [14], PaDiM [16], CFA [31], CFlow [20] and MSFlow [11]. Since these methods are all built on 2D images, we evaluate them using RGB images in the TA dataset. The results presented in Table 1 demonstrate the exceptional performance of DCNF on the TA dataset. For instance, DCNF showed a 3.67% better AUROC than MSFlow, which achieved the second best AUROC on TA. Figure 6 exhibits the visualization of DCNF on the TA dataset.

In order to showcase the effectiveness of our fusion strategy, we also compare the outcomes obtained from the RGB branch, depth branch, and the fusion of the two branches in Table 2. The results demonstrate that the dual-branch cross-fusion strategy we proposed could effectively fuse the RGB images and depth maps through dual-branch normalizing flow.

**Figure 7:**
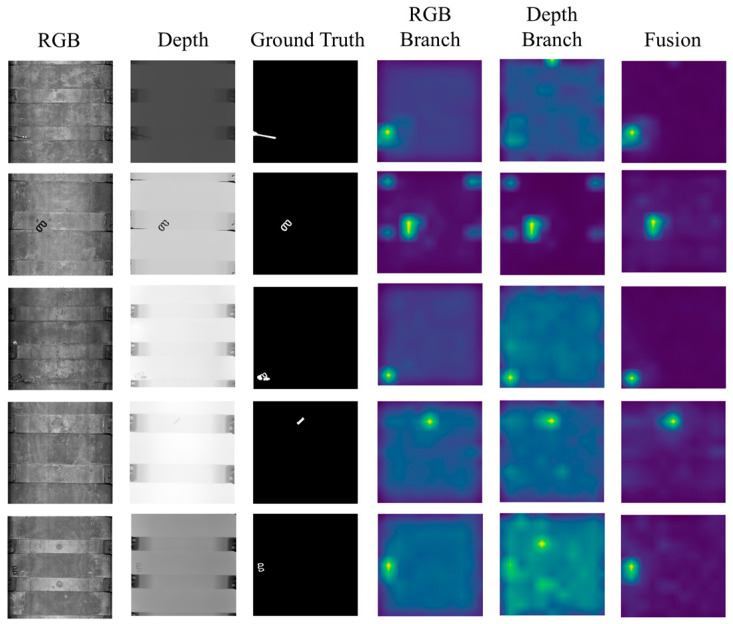
Comparison of the visualization results of different branches.

To demonstrate that the dual-branch network is able to take full advantage of both RGB images and depth maps, we present visualizations of each branch. The results are compared with the fusion results shown in Figure 7. The visualization results show that the fused results have less noise than each branch of the DCNF.

In industrial applications, we pay more attention to the detection rate of anomalies. Therefore, we evaluate the recall of DCNF on the TA dataset in Table 3. Notably, the DCNF outperforms previous methods and achieves a 2.17% better recall than the second option. Considering that our goal is to judge whether a track is abnormal or not, we believe it is essential to employ recall as an evaluation metric.

Table 4 presents a recall performance comparison of the RGB branch, the depth branch, and their fusion using the DCNF framework on the TA dataset. The results demonstrate that the fusion strategy significantly outperforms individual modalities, achieving a 96.47% recall score, which is highlighted as the best performance.

The RGB branch alone achieves 94.13% recall, slightly surpassing the depth branch (93.85%). While both unimodal branches deliver strong results, the marginal superiority of the RGB branch suggests that visual features (e.g., color, texture) may provide slightly more discriminative cues for the task on this dataset. However, the depth branch’s competitive performance (93.85%) underscores the importance of geometric and spatial information in enhancing recognition robustness. The fused result (96.47%) exhibits a notable improvement over both unimodal branches, with gains of +2.34% over RGB and +2.62% over depth. This indicates strong complementary characteristics between RGB and depth modalities, where their combination effectively mitigates individual limitations (e.g., RGB sensitivity to lighting variations, depth ambiguity in textureless regions). The fusion mechanism in DCNF successfully leverages cross-modal synergies to achieve state-of-the-art performance.

### 4.4. Ablation Study

We here investigate the influence of individual components of DCNF, including adding different modules, the number of the normalizing flow blocks, different extractions, and the K set in TopK.

#### 4.4.1. Influence of Different Modules

The results in Table 5 show a great improvement in AUROC using both MP and the Fusion Flow module. As can be seen, both MP and the Fusion Flow module have a lifting effect on the dual-branch normalizing flow.

By separating the modal input, it can be verified whether multi-modal fusion is significantly better than single-modal detection, and the complementarity of the shape information provided by the depth map (such as the three-dimensional structure of a foreign object) and the RGB texture information is proven. The experimental results show that the fusion strategy can effectively improve the robustness of complex anomalies (such as the detection of small-scale foreign bodies and illumination changes) by combining the advantages of the two modes.

#### 4.4.2. Influence of the Number of Normalizing Flow Blocks

Existing methods focus on stacking as many normalizing flow blocks as they can, but the increasing number of blocks will raise the amount of computation. Therefore, we here investigate an optimal set by conducting experiments with varying numbers of normalizing flow blocks. The utilization of a greater number of normalizing flow blocks, as demonstrated in Table 6, can enhance the accuracy of the model. It is worth noting that the accuracy when using 8 is same as when using 10, so we adopt n=8 in all our experiments.

#### 4.4.3. Influence of Different Extractions

In this subsection, we experiment in different feature extractions including ResNet-18 (RN-18), ResNet-50 (RN-50) and WideResNet-50 (WRN-50), and compare the results with those from the prior methods shown in Table 7. The results demonstrate that our DCNF achieves a significant improvement over the prior methods, even with a lightweight RN-18. The WRN-50 layers three features based on ImageNet pre-training balance semantic expression and spatial details, combined with average pooled down-sampling to reduce computational complexity and improve inference efficiency while maintaining high accuracy.

However, the features of WRN-50 pre-trained on ImageNet may exhibit sensitivity to domain differences (e.g., medical images, satellite images), thus necessitating the implementation of additional fine-tuning or domain adaptation strategies. Therefore, subsequent work may be appropriately fine-tuned for railway applications.

#### 4.4.4. Influence of the *K* Sets in *TopK*:

**Figure 8:**
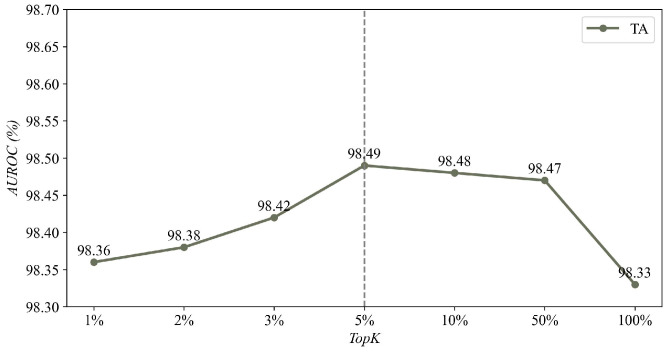
Ablation study of AUROC in % with different *K* sets of *TopK* on the TA dataset. The best result is plotted in the figure.

As a hyperparameter in the post-processing stage, we have conducted an ablation study to determine the optimal K. Different from the other methods that compute the anomaly scores in the whole images, we select only TopK pixels for the calculation of anomaly scores, which helps to minimize the interference of noise. The results of these experiments are presented in Figure 8. TopK represents the ratio of the maximum scores of pixels in the image; the specific formula is $K\times H_{img}\times W_{img}$ and the average score gives special solutions when TopK is set as $100\%\times H_{img}\times W_{img}$. As shown in Figure 8, when the TopK is set to 5%, the accuracy of DCNF achieves the best value in most categories.

It is noteworthy that when the TopK is set as $100\%\times H_{img}\times W_{img}$, the accuracy will obviously decrease. According to our analysis, this is attributed to the fact that in the final anomaly map, only the anomaly exhibits a significantly high anomaly score. Consequently, when dealing with images containing minor anomalies, calculating the average of the anomaly scores for the entire image results in a lower anomaly score.

## 5. Conclusions

In this paper, we have proposed a novel dual-branch cross-fusion strategy for RGB-D anomaly detection in combination with depth maps to take advantage of the shape information. Specifically, we propose a mutual perception module and fusion flow module to utilize the cross-complementary prior knowledge and the different information given by the dual-branch module. The proposed DCNF achieves state-of-the-art performance on the TA dataset, thereby demonstrating the effectiveness of the DCNF. Additionally, we explored the effects of different K sets of TopK on accuracy, and we have concluded that for images with small anomalies, smaller K sets are more helpful to overall detection. Future work could explore adaptive fusion weights to further optimize modality contributions under varying scenarios, as well as investigating whether the depth branch’s marginally lower standalone performance stems from inherent data limitations (e.g., noise in depth sensors) or architectural constraints in feature extraction. We hope our work will inspire future research on industrial anomaly detection.

## Figures and Tables

**Table 1:** Comparison of area under ROC (AUROC) in % of different methods on the TA dataset. The best is highlighted in bold, and the second is highlighted with underlining.

Method	GANomaly [13]	PaDiM [15]	CFA [31]	CFlow [19]	MSFlow [10]	DCNF
AUROC	80.30	91.70	94.28	93.12	94.75	**98.49**

**Table 2:** AUROC scores (%) compared for RGB branch, depth branch, and the fusion of the two branches on the TA dataset. The best is highlighted in bold, and the second is highlighted with underlining.

Method	DCNF (RGB)	DCNF (Depth)	DCNF
AUROC	97.02	95.94	**98.49**

**Table 3:** The recall score (%) for the TA dataset compared with previous methods. The best is highlighted in bold, and the second best is highlight with underlining.

Method	GANomaly	PaDiM	CFA	CFlow	MSFlow	DCNF
Recall	83.40	90.81	94.30	93.99	92.93	**96.47**

**Table 4:** A comparison of recall scores (%) for the RGB branch, the depth branch, and the fusion of the two branches on the TA dataset. The best is highlighted in bold, and the second best is highlight with underlining.

Method	DCNF (RGB)	DCNF (Depth)	DCNF
Recall	94.13	93.85	**96.47**

**Table 5:** Ablation study of AUROC in % on the TA dataset when adding different modules. The best is highlighted in bold.

MP	Fusion Flow	TA
×	×	97.26
×	√	97.76
√	×	98.08
√	√	**98.49**

**Table 6:** Ablation study of AUROC in % with the number of normalizing flow blocks n on the TA dataset. Bold values denote the best result on the TA dataset.

*n*	2	5	8	10
AUROC	97.76	97.87	**98.49**	**98.49**

**Table 7:** Comparison study of AUROC in % with different extractions. The best is highlighted in bold, and the second best is highlighted with underlining.

Method	GANomaly	PaDiM	CFA	CFlow	MSFlow	DCNF (RN-18)	DCNF (RN-50)	DCNF (WRN-50)
TA	80.3	91.7	94.28	93.12	94.75	97.79	98.13	**98.49**

## Data Availability

The generated data supporting the findings are currently not publicly accessible, but may be obtained from the authors upon reasonable request.

## Abbreviations

The following abbreviations are used in this manuscript:

AE	Autoencoder
AUROC	Area Under Receiver Operator Curve
DCNF	Dual-Branch Cross-Fusion Normalizing Flow
TA	Track Anomaly
MP	Mutual Perception module
GAN	Generative Adversarial Network
kNN	k-Nearest Neighbor
NF	Normalizing Flow
RN-18	ResNet-18
RN-50	ResNet-50
WRN-20	WideResNet-50
